# A wheat caffeic acid 3-O-methyltransferase TaCOMT-3D positively contributes to both resistance to sharp eyespot disease and stem mechanical strength

**DOI:** 10.1038/s41598-018-24884-0

**Published:** 2018-04-25

**Authors:** Minxia Wang, Xiuliang Zhu, Ke Wang, Chungui Lu, Meiying Luo, Tianlei Shan, Zengyan Zhang

**Affiliations:** 1 The National Key Facility for Crop Gene Resources and Genetic Improvement, Institute of Crop Science, Chinese Academy of Agricultural Sciences, Beijing, 100081 P.R. China; 2 School of Animal, Rural and Environmental Sciences, Nottingham Trent University, Brackenhurst Campus, Nottingham, NG250QF United Kingdom

## Abstract

Plant caffeic acid 3-O-methyltransferase (COMT) has been implicated in the lignin biosynthetic pathway through catalyzing the multi-step methylation reactions of hydroxylated monomeric lignin precursors. However, genetic evidence for its function in plant disease resistance is poor. Sharp eyespot, caused primarily by the necrotrophic fungus *Rhizoctonia cerealis*, is a destructive disease in hexaploid wheat (*Triticum aestivum* L.). In this study, a wheat COMT gene *TaCOMT-3D*, is identified to be in response to *R*. *cerealis* infection through microarray-based comparative transcriptomics. The *TaCOMT-3D* gene is localized in the long arm of the chromosome 3D. The transcriptional level of *TaCOMT-3D* is higher in sharp eyespot-resistant wheat lines than in susceptible wheat lines, and is significantly elevated after *R*. *cerealis* inoculation. After *R*. *cerealis* inoculation and disease scoring, *TaCOMT-3D*-silenced wheat plants exhibit greater susceptibility to sharp eyespot compared to unsilenced wheat plants, whereas overexpression of *TaCOMT-3D* enhances resistance of the transgenic wheat lines to sharp eyespot. Moreover, overexpression of *TaCOMT-3D* enhances the stem mechanical strength, and lignin (particular syringyl monolignol) accumulation in the transgenic wheat lines. These results suggest that *TaCOMT-3D* positively contributes to both wheat resistance against sharp eyespot and stem mechanical strength possibly through promoting lignin (especially syringyl monolignol) accumulation.

## Introduction

Hexaploid wheat (*Triticum aestivum* L., namely bread wheat or common wheat, genome AABBDD) is one of the most widely cultivated and consumed food crops worldwide. Stem lodging in wheat is a major agronomic problem that has far-reaching economic consequences^1^. Crop lodging is a complicated phenomenon that is influenced by many factors from physiology and genetics to husbandry and environment^2,3^. Green Revolution has effectively increased lodging resistance and the harvest index through the use of semi-dwarf trait. However, a reduction in plant height for lodging resistance may reduce the photosynthetic capacity of a canopy which would then limit the final yield^4^. A paper reported that the optimum plant height for maximum photosynthetic capacity is between 70 cm and 100 cm in wheat^5^. Thus, alternative strategies for further improvement in lodging resistance of crops must be developed. Increasing the stem/culm mechanical strength has been shown to be efficient for improving crop lodging resistance^2,3,6^. Sharp eyespot is a devastating soil-borne fungal disease globally, which results in yield losses of 10–40%^7,8^. The necrotrophic fungus *Rhizoctonia cerealis* Van der Hoeven is a major causal pathogen of sharp eyespot^9^. The pathogen can infect the basal stems and sheath, destroy the transport tissues of infected plants and block transportation of substances required for nutrition, leading to lodging and dead spikes. Isolation and application of important resistance-related genes make it possible to generate wheat materials with resistance to both this disease and lodging.

Lignin is a phenolic cell wall polymer, which is composed of guaiacyl, syringyl and p-hydroxyphenyl units derived from the monolignol precursors (p-coumaryl, coniferyl, and sinapyl alcohols), respectively (Supplemental Fig. S1)^10–12^. Lignin is mainly deposited in the walls of certain specialized cells such as the tracheary elements, sclerenchyma and phloem fibers^13^. It plays pivotal roles in cell wall structural integrity, stem strength, water transport, mechanical support, and responses to various environmental and biotic stresses^12–18^. During plant-pathogen interactions, the deposition of lignin is thought to play a role as a physical barrier against infection. Lignification also can chemically modify cell walls to be more resistant to cell wall-degrading enzymes, increase the resistance of cell walls to the diffusion of toxins from the pathogens to the hosts and of nutrients from the hosts to the pathogens, produce toxic precursors and free radicals, and entrap the pathogens^11,19^. Monolignol biosynthesis genes contribute to local resistance to biotrophic and hemibiotrophic pathogens. For example, in diploid wheat (*Triticum monococcum*), transient knock-down of monolignol pathway enzymes [phenylalanine ammonia-lyase (PAL), caffeoyl-CoA O-methyltransferase (CCoAOMT), caffeic acid 3-O-methyltransferase (COMT) and cinnamyl alcohol dehydrogenase (CAD)] individually and pair-wise led to decreased basal immunity or penetration resistance to the fungal pathogens *Blumeria graminis* f. sp. *tritici* and *Blumeria graminis* f. sp. *Hordei*, respectively^11^. In rice (*Oryza sativa* L.), the cinnamoyl-CoA reductase (CCR) contributed to R-mediated immunity against the hemibiotrophic fungal pathogen *Magnaporthe grisea*^20,21^. In Arabidopsis and tobacco, loss or down-regulation of *PAL* led to decreased basal immunity to the hemibiotrophic bacterial pathogen *Pseudomonas syringae*^22^ and to the biotrophic viral pathogen tobacco mosaic virus^23,24^, respectively. In *Arabidopsis*, through assessment on double mutant (*cad*-*C cad*-*D*), the genetic and functional analysis indicated that the *CAD*-*C* (At3g19450) and *CAD*-*D* (At4g34230) were the primary genes being involved in lignin biosynthesis^25,26^, and acted as essential components of defense response against virulent and avirulent strains of the bacterial pathogen *Pseudomonas syringae* pv. *tomato*^16^. Recently, Rong *et al*. reported that the wheat CAD named TaCAD12 positively contributed to host defense response to a necrotrophic fungal pathogen *R*. *cerealis*^27^. More recent paper reported that a maize (*Zea mays* L.) CCoAOMT named *ZmCCoAOMT2* was isolated^18^. *ZmCCoAOMT2* has been shown to confer quantitative resistance to both southern leaf blight and gray leaf spot through altering lignin level and other metabolites of the phenylpropanoid pathway and regulation of programmed cell death^18^. However, defense role of COMT in plant species is poorly understood.

The COMT is an O-methyltransferase that can potentially act in various branches of the phenylpropanoid pathway^12,13^. In gymnosperms, the COMT is believed to catalyze caffeic acid rather than 5-hydroxyferulic acid. In angiosperms, COMT can catalyze caffeic acid and 5-hydroxyferulic acid as well flavone^13,28,29^. The highly conserved S-adenosyl methionine (SAM) binding domain in COMT proteins indicates the use of SAM as the methyl group donor to the hydroxyl group of a methyl acceptor molecule^30^. COMT catalyzes the multi-step methylation reactions of hydroxylated monomeric lignin precursors^11,12,31,32^. COMT is involved in the methylation of caffeic acid to ferulic acid, which is then hydroxylated at position five by ferulate-5-hydroxylase. The subsequent methylation by COMT at this position yields sinapic acid. Similarly, COMT catalyzes the 3-O-methylation of caffeoyl aldehyde and conifer-aldehyde/coniferyl alcohol in the process of guaiacyl and syringyl monolignol biosyntheses^11,12^. For example, Alfalfa COMT (MsCOMT) exhibits the higher catalyzing efficiency towards 5-hydroxyconiferaldehyde and caffeoyl aldehyde rather than caffeic acid^33^. In *Brachypodium distachyon*, COMT has high affinity for a variety of substrates including flavonoid compounds, with the greatest activity towards caffeic acid and caffeoyl aldehyde^34^. Interestingly, *TaCM* (GenBank accession number EF413031), a wheat *COMT* gene, was reported to locate on wheat chromosome 3BL and to encode an authentic COMT enzyme TaCM^2,13^. Kinetic analysis indicated that the TaCM protein exhibited the highest catalyzing efficiency towards caffeoyl aldehyde and 5-hydroxyconiferaldehyde as substrates, suggesting a pathway leading to syringyl lignin via aldehyde precursors^13^. Antisense *TaCM* transgenic tobacco assay indicated that the down-regulation of *TaCM* resulted in reduction in COMT activity and a sharp reduction in the syringl monolignol^13^. It has been demonstrated that in maize, rice and sorghum (*Sorghum bicolor* L.), the O-methyltransferase responsible for the production of syringyl lignin was also involved in the synthesis of lignin-linked tricin^35^. Several groups aimed to improve the production of biofuel and generated transgenic plants with deficit/reduction of syringyl lignin unit through knock-out/down of the lignin-related *COMT* genes in plants^13,36–39^. However, no genetic evidence for defense roles of COMT genes has yet been reported in plants.

In this study, the probe with Agilent GeneChip number A_99_P198406, being homologous to certain plant COMTs, was identified to be in response to *R*. *cerealis* R0301 through comparative transcriptomic analysis on the sharp eyespot-resistant wheat line CI12633 and the susceptible wheat line Wenmai 6. Subsequently, this *COMT* gene located on wheat chromosome 3DL, designated as *TaCOMT-3D*, was cloned from the resistant wheat line CI12633. Importantly, through generation and evaluation of *TaCOMT-3D*- silencing and overexpressing wheat plants, we dissected roles of *TaCOMT-3D* in resistance response to *R*. *cerealis*, lignin accumulation, and stem mechanical strength in wheat. The functional characterization proved that *TaCOMT-3D* positively contributed to syringl monolignol biosynthesis, wheat resistance response to *R*. *cerealis*, and the stem mechanical strength.

## Results

### Identification, cloning, and chromosomal location of *TaCOMT-3D*

**Figure 1:**
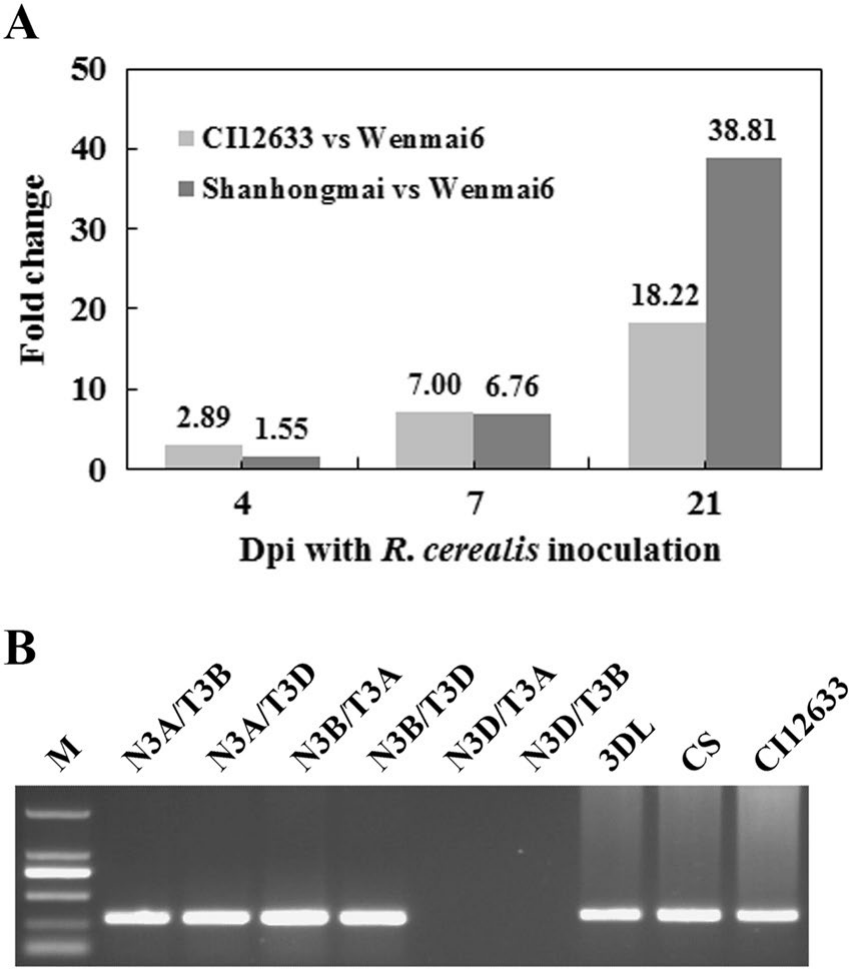
Identification and chromosome location of *TaCOMT-3D*. (**A**) The FoldChange of *TaCOMT-3D* transcriptional level derived from microarray analysis (GEO accession number GSE69245) between the *Rhizoctonia cerealis*-resistant wheat line CI12633/Shanhongmai and susceptible wheat cultivar Wenmai 6 at 4, 7, and 21 days post inoculation (dpi) with *R*. *cerealis*. **(B)** Chromosome location of *TaCOMT-3D* using nullitetrasomic and double ditelosomic lines derived from wheat cultivar Chinese Spring (CS). Marker, DL2, 000 DNA marker; N3A/T3B, N3A/T3D, N3B/T3A, N3B/T3D, N3D/T3A, N3D/T3B, six nulli-tetrasomic lines; 3DL, di-telosomic line of chromosome 3B.

Microarray-based comparative transcriptomic assay was used to identify differentially expressed probes between sharp eyespot-resistant wheat line CI12633/Shanhongmai and the susceptible wheat line Wenmai 6 inoculated with *R*. *cerealis* R0301 (microarray raw data, GEO accession number GSE69245). Among them, the probe A_99_P198406, 100% matching to 3′-terminal sequence of a wheat cDNA sequence with accession number AK332908, displayed significantly transcriptional increase in sharp eyespot-resistant wheat lines (CI12633 and Shanhongmai) than in the susceptible wheat line Wenmai 6 at 4, 7, and 21 dpi (day post inoculation) with *R*. *cerealis* R0301 (Fig. 1A), respectively. For example, the probe A_99_P198406 showed 2.89-, 7.00-, and 18.22-fold transcriptional increase in CI12633 than in Wenmai 6 at 4, 7, and 21 dpi, respectively. The full-length cDNA sequence of the gene, containing the complete open-reading frame (ORF), was cloned from cDNA of CI12633 stems inoculated with *R*. *cerealis* R0301 using nested PCR method and two pairs of primers designed based on the sequence of AK332908. The pairwise alignment showed that only one nucleotide difference exists between the cloned cDNA sequence of the gene from CI12633 and the corresponding sequence of AK332908. The gene promoter sequences were also cloned from CI12633 and Wenmai 6, respectively. The sequence analysis indicated that no difference existed between the gene promoter sequences from these two wheat lines (Supplemental Fig. S2).

To clarify the chromosome assignment of this gene, the cloned genomic sequence was subjected to alignment with the chromosome-based draft sequence of the common wheat (Chinese Spring) genome (http://www.wheatgenome.org/) (IWGSC). The alignment result displayed that this gene sequence shared 95.33%, 95.42%, and 100% identities with the matched sequences of the TGACv1_scaffold_195106_3AL, TGACv1_scaffold_220646_3B, and TGACv1_scaffold_249256_3DL, respectively. The data suggested that the gene should locate on the long arm of the wheat chromosome 3D. To verify this alignment on the chromosomal localization, genomic DNAs extracted from Chinese Spring Nulli-tetrasomic lines (NTs) were used as template of PCR using the gene-specific primers to confirm the chromosome location of the gene. The results proved that the gene was indeed located on the long arm of chromosome 3D (3DL) (Fig. 1B). Blast search against the nucleotide acid sequence database in the National Center for Biotechnology Information (NCBI) indicated that the gene sequence is homologous to COMT. Thus, this gene cloned from CI12633 was designated as *TaCOMT-3D*.

### Sequence characteristics of TaCOMT-3D

**Figure 2:**
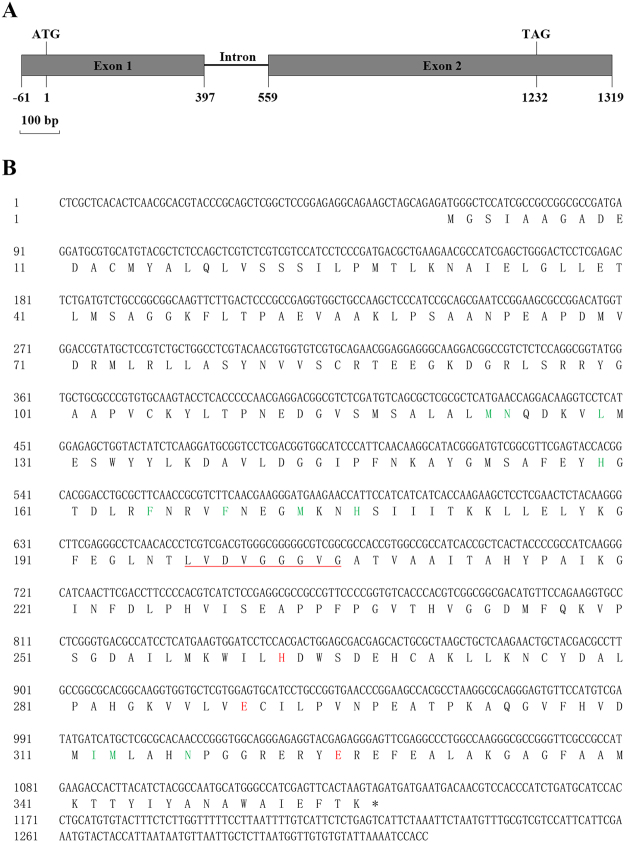
Gene structure and deduced amino acid sequence of *TaCOMT-3D*. (**A**) Gene structure of *TaCOMT-3D*. Dark grey portions and full line represent exons and introns, respectively. **(B)** The deduced amino acid sequence of *TaCOMT-3D*. Conserved motif for SAM binding is marked by underline, catalytic residues are labeled as red, and active site substrate binding/positioning residues are labeled as green.

**Figure 3:**
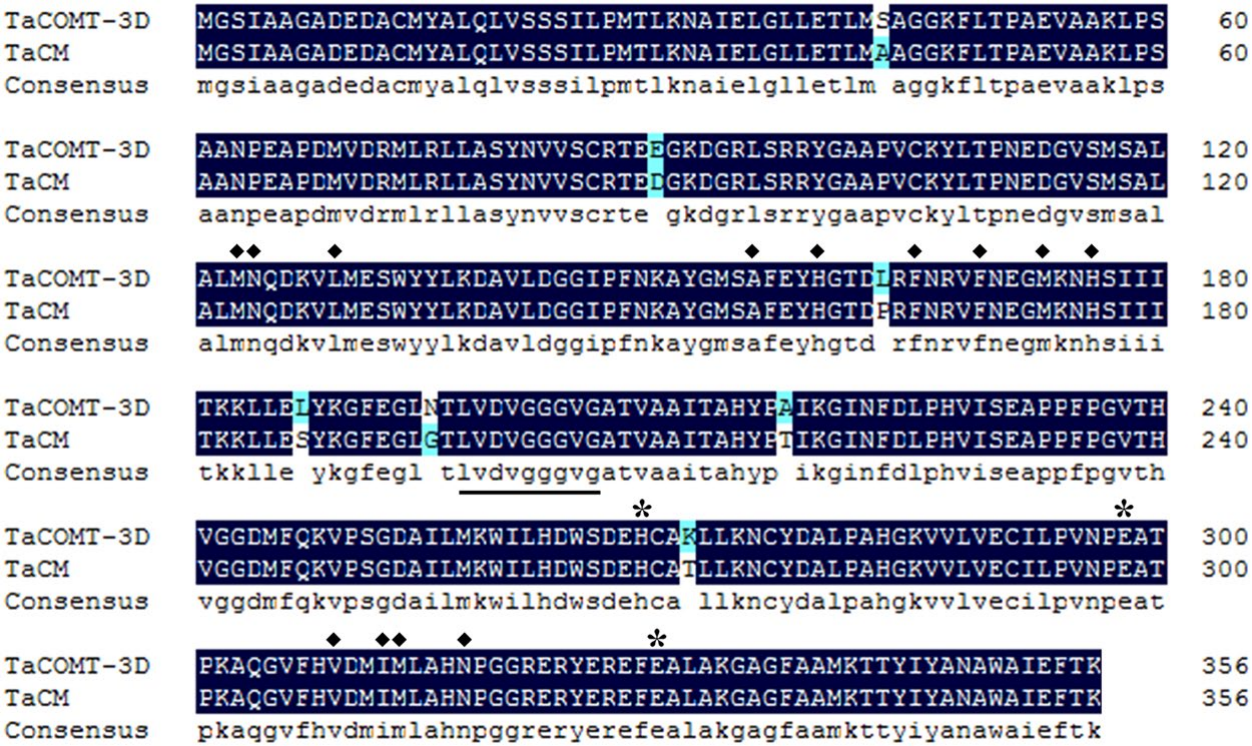
Sequence alignment of TaCOMT-3D and another Caffeic acid 3-O-methyltransferase TaCM from wheat. The software DANMAN was used to perform the sequence alignment. Conserved motif for SAM binding is marked by underline, catalytic residues are marked by asterisk, and active site substrate binding/positioning residues are marked by diamond.

The full-length cDNA and genomic sequences of *TaCOMT-3D* was cloned from cDNA and genomic DNA of CI12633 stems inoculated with *R*. *cerealis* R0301, respectively. The comparison of the cDNA and genomic sequences indicated that *TaCOMT-3D* genomic sequence with 1480 bp-length was comprised of one intron (161-bp length) and 2 exons (Fig. 2A). The *TaCOMT-3D* cDNA sequence with 1319-bp contains an ORF with 1071-bp (from 62 to 1132 nt), the 5′-untranslated region (UTR) with 61-bp, and 3′-UTR with 187-bp (Fig. 2A,B). Blast results showed that *TaCOMT-3D* was the closest to the previously reported wheat caffeic acid 3-O-methyltransferase encoding gene *TaCM* (GenBank accession no. EF413031; Ma and Xu, 2008)^13^ and flavonoid O-methyltransferase encoding gene *TaOMT2* (GenBank accession no. DQ223971)^28,29^. A pairwise alignment indicated that the *TaCOMT-3D* cDNA sequence shared 95.24% and 95.33% identities with *TaCM* and *TaOMT2*. The predicted protein TaCOMT-3D is consisted of 356 amino acids with a molecular weight of 38.697 kD and a theoretical iso-electric point of 6.042. As shown in Fig. 2B, the TaCOMT-3D protein contains one dimerization domain (27–78 aa) and one methyltransferase domain (121–341 aa). The comparison of amino acid sequences showed that the TaCOMT-3D protein shared a 98.03% identity with the wheat flavonoid O-methyltransferase TaOMT2 and caffeic acid 3-O-methyltransferase TaCM (GenBank accession no. ABB03907.1). It was reported that only one amino acid difference existed between TaCM and TaOMT2 proteins, in which E89 in TaCM was replaced by D89 in TaOMT2^13^. As shown in Fig. 3, seven amino acid differences existed between the protein sequences of TaCOMT-3D and TaCM. Fortunately, TaCOMT-3D and TaCM proteins shared 100% identity in all the biochemically functional sites, including the SAM binding motif (LVDVGGGVG), catalytic residues (H269, E297 and E329), and active site substrate binding/positioning residues (M123, N124, L129, A155, H159, F165, F169, M173, H176, V309, I312, M313, and N317) (Fig. 3). In the previous paper, both enzymatic *in vitro* and *in vivo* (transgenic tobacco) results revealed that TaCM was an authentic COMT enzyme and participated predominantly in syringl monolignol biosynthesis^13^. Thus, TaCOMT-3D should be an authentic COMT enzyme, too.

### The expression profile of *TaCOMT-3D*

**Figure 4:**
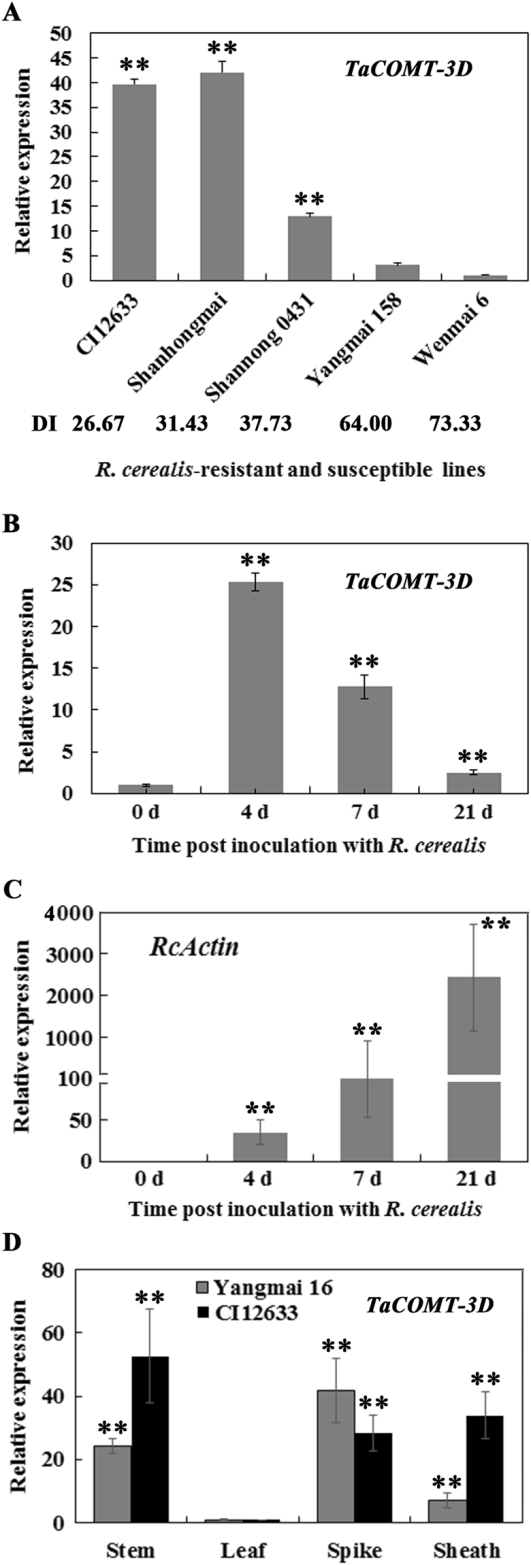
Transcription of *TaCOMT-3D* in wheat (*Triticum aestivum*). (**A**) Expression patterns of *TaCOMT-3D* in five wheat cultivars with different degrees of resistance to *Rhizoctonia cerealis*. The expression level of *TaCOMT-3D* in the Wenmai 6 plants inoculated with *R*. *cerealis* for 21 days was set to 1. DI indicates disease index of sharp eyespot. **(B)** qRT-PCR analysis of *R*. *cerealis Actin* (*RcActin*) gene in CI12633 plants inoculated with *R. cerealis* at the indicated time. **(C)** qRT-PCR analysis of *TaCOMT-3D* induction by *R*. *cerealis* inoculation in CI12633 plants. The expression level of *TaCOMT-3D* at 0 day post inoculation (dpi) with *R*. *cerealis* is set to 1. **(D)** Transcription of *TaCOMT-3D* in roots, stems, leaves, sheaths, and spikes of Yangmai 16 and CI12633 plants. The transcriptional level of *TaCOMT-3D* in leaves was set to 1. Statistically significant differences are derived from the results of three independent replications (*t*-test: **P < 0.01).

To investigate if the gene expression is related with sharp eyespot-resistance, quantitative real-time PCR (qRT-PCR) technique was used to analyze the transcriptional levels of *TaCOMT-3D* in stems of five wheat lines/cultivars with different resistance degrees, including sharp eyespot-resistant lines CI12633 and Shanhongmai, moderately-resistant line Shannong 0431, moderately-susceptible wheat cultivar Yangmai 158, and susceptible cultivar Wenmai 6 at 21 dpi with *R*. *cerealis* R0301. The results showed that *TaCOMT-3D* transcriptional level was the highest in Shanghongmai, slightly decreased in CI12633, gradually declined in Shannong 0431 and Yangmai 158, and reached the lowest in Wenmai 6 (Fig. 4A). The data indicated that the *TaCOMT-3D* transcriptional level is associated with the resistance degree of wheat against sharp eyespot caused by *R*. *cerealis*. Additionally, qRT-PCR analysis suggested that the *R*. *cerealis* biomass (represented by the expression level of *R*. *cerealis Actin* gene) increased with the pathogen inoculation duration (Fig. 4B), and the expression level of *TaCOMT-3D* in CI12633 was markedly elevated after infection of *R*. *cerealis* (Fig. 4C). Furthermore, the tissue expression profile of *TaCOMT-3D* at inflorescence stage was investigated in wheat cultivars Yangmai 16 and CI12633. As shown in Fig. 4D, at the inflorescence stage, the expression level of *TaCOMT-3D* was the highest in the inflorescence and stem tissues in both the wheat lines Yangmai 16 and CI12633, respectively, and the lowest in the leaf tissue in both Yangmai 16 and CI12633 at 21 dpi with *R*. *cerealis* R0301. These results suggested that *TaCOMT-3D* might participate in wheat defense response to *R*. *cerealis* infection.

### Knock-down of *TaCOMT-3D* suppresses host resistance to sharp eyespot

**Figure 5:**
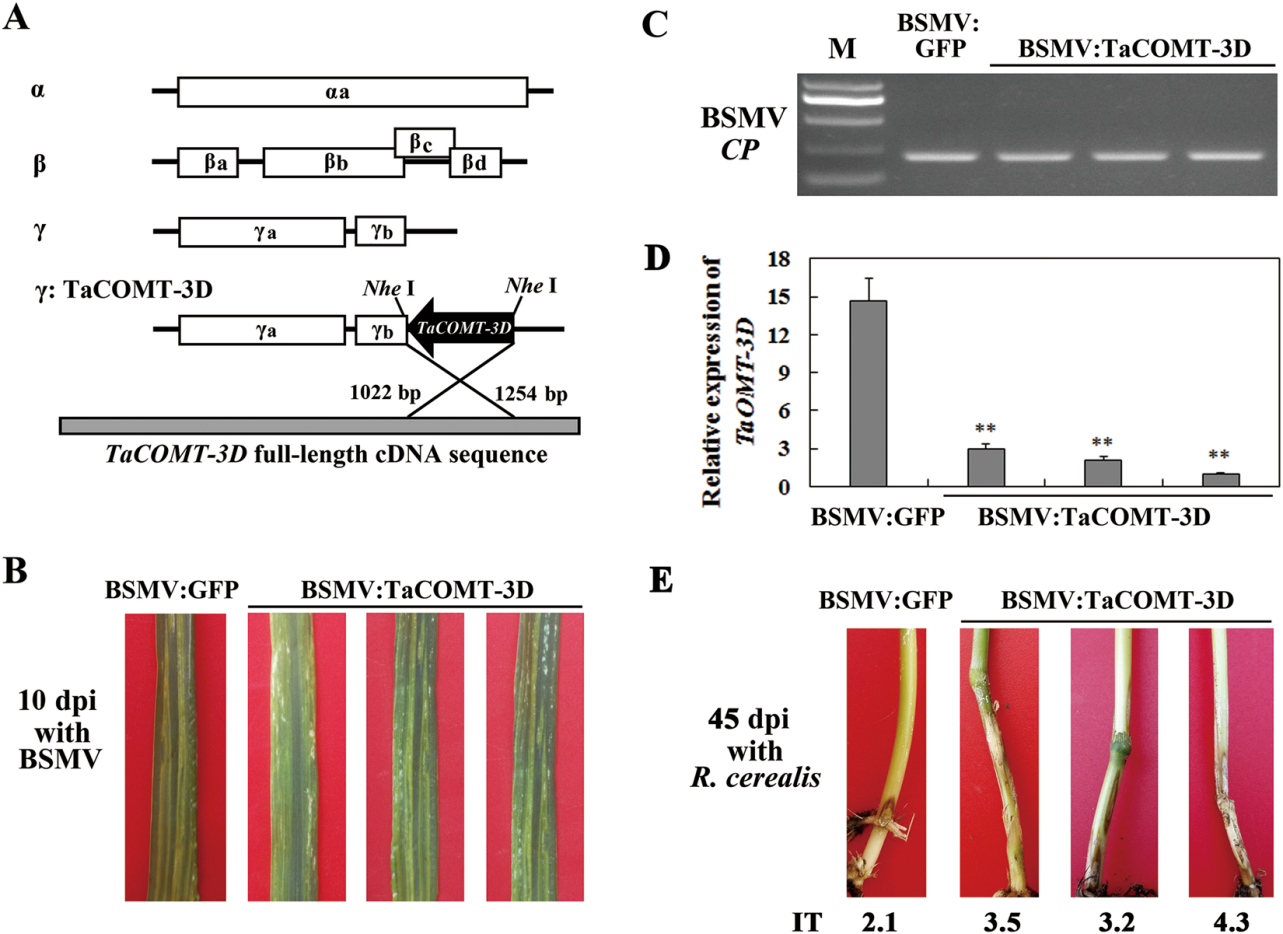
Silencing of *TaCOMT-3D* by barley stripe mosaic virus (BSMV)-induced gene silencing impairs CI12633 resistance to *Rhizoctonia cerealis*. (**A**) Scheme of genomic RNAs of BSMV construct and the construct of the recombinant virus expressing the wheat (*Triticum aestivum*) gene *TaCOMT-3D*, BSMV:TaCOMT-3D. The orientation of the *TaCOMT-3D* insert is indicated by dark boxes. (**B**) Mild chlorotic mosaic symptoms were observed on leaves at 10 days post inoculated (dpi) with BSMV: GFP or BSMV:TaCOMT-3D. (**C**) RT-PCR analysis of the transcription levels of BSMV coat protein encoding gene *CP* in the wheat plants infected by BSMV: GFP or BSMV:TaCOMT-3D at 10 dpi with *R*. *cerealis*. (**D**) qRT-PCR analysis of the transcription levels of wheat *TaCOMT-3D* gene in the wheat plants infected by BSMV: GFP or BSMV:TaCOMT-3D at after infection with *R*. *cerealis*. The relative transcript level of *TaCOMT-3D* in BSMV:TaCOMT-3D-infected (*TaCOMT-3D*-silencing) wheat CI12633 plants is relative to that in BSMV: GFP-infected (control) plants (set to 1). Significant differences were analyzed based on three replications (*t*-test: **P < 0.01). Error bars indicate standard deviation. (**E**) Sharp eyespot symptoms of the control and *TaCOMT-3D*-silencing CI12633 plants at 45 dpi with *R*. *cerealis*. IT indicates the infection type of wheat plant response to *R*. *cerealis*.

The barley stripe mosaic virus (BSMV)-based virus-induced gene silencing (VIGS) technique was used to analyze rapidly the functions of *TaCOMT-3D *in wheat. To knockdown *TaCOMT-3D* expression in the resistant wheat line CI12633 through BSMV-VIGS method, a 3′-terminal fragment (233-bp) specific to *TaCOMT-3D* was inserted in an antisense orientation into *Nhe* I restriction site of the BSMV RNAγ chain to generate BSMV:TaCOMT-3D recombinant construct (Fig. 5A). When the sharp eyespot-resistant wheat CI12633 plants had been infected with BSMV for 10 d, BSMV-infected symptom was present in both BSMV:GFP- and BSMV:TaCOMT-3D-inoculated CI12633 plants (Fig. 5B), and the expression of BSMV *CP* gene could be detected from stems of these plants (Fig. 5C). The results indicated that these BSMV:GFP and BSMV:TaCOMT-3D viruses successfully infected these viruses-inoculated wheat plants. qRT-PCR analysis indicated that the *TaCOMT-3D* transcriptional level was substantially reduced in stems of BSMV:TaCOMT-3D-infected CI12633 plants, revealing that *TaCOMT-3D* was successfully knock-downed in BSMV:TaCOMT-3D-infected CI12633 plants (Fig. 5D). Then, these plants were further inoculated with *R*. *cerealis* isolate WK207 to evaluate the defense role of *TaCOMT-3D*. At 45 dpi with *R*. *cerealis* WK207, more serious symptom of sharp eyespot was present on the stems of BSMV:TaCOMT-3D-infected CI12633 plants (infection types: 3.5, 3.2, 4.3), compared with that on the stems of BSMV:GFP-treated CI12633 plants (average infection type: 2.1) (Fig. 5E). These results proved that the silence of *TaCOMT-3D* in CI12633 suppressed host resistance to sharp eyespot caused by *R*. *cerealis*, suggesting that *TaCOMT-3D* is required for the wheat resistance against *R*. *cerealis* infection.

### *TaCOMT-3D* overexpression improves wheat resistance to sharp eyespot

**Figure 6:**
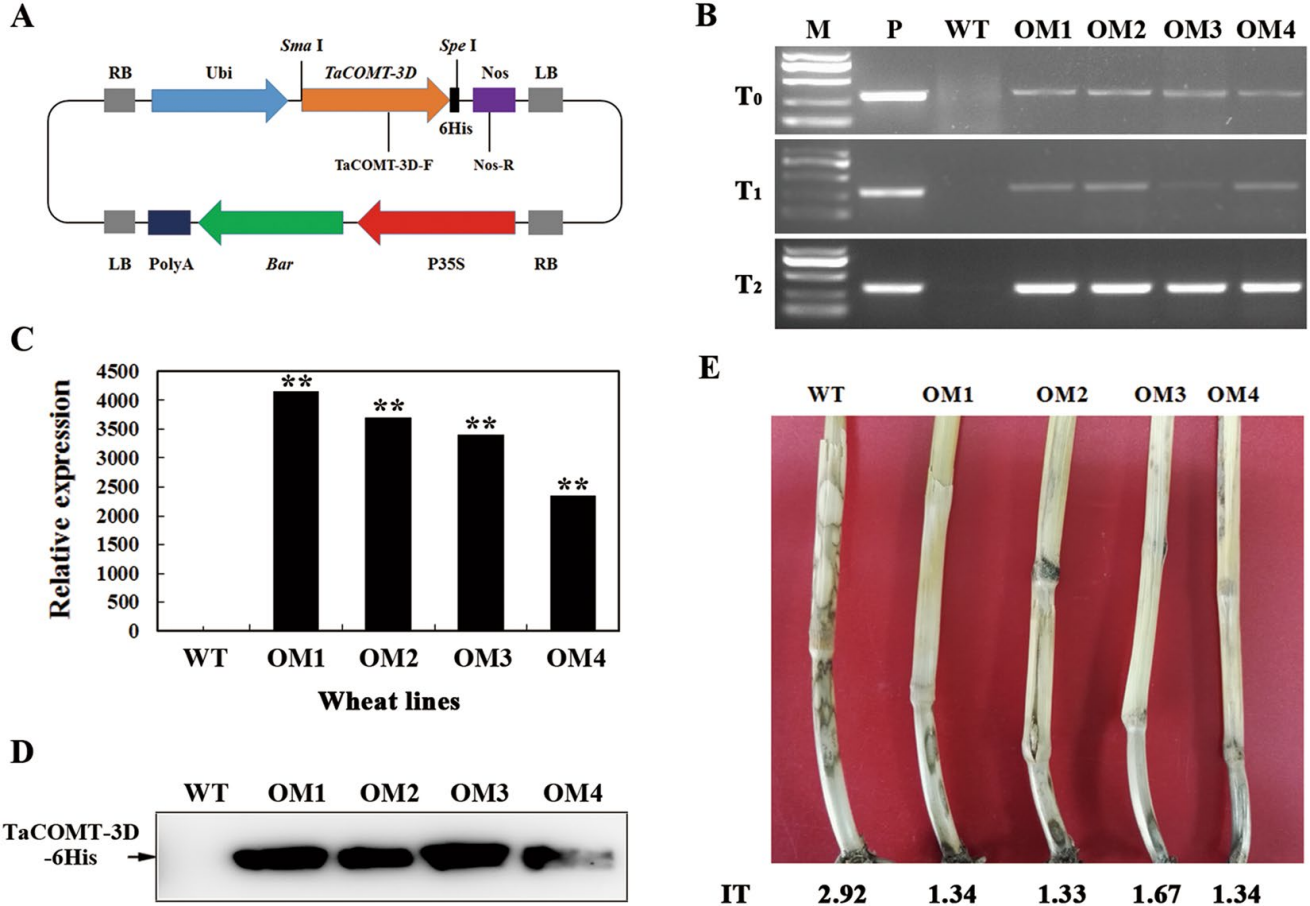
Molecular characterizations of *TaCOMT-3D*-overexpressing wheat plants and responses to *Rhizoctonia cerealis* infection. (**A**) The diagram of *TaCOMT-3D* overexpressing transformation vector pWMB-TaCOMT-3D. **(B)** PCR pattern of *TaCOMT-3D*-overexpressing transgenic lines in T_0_-T_2_ generations and wild-type (WT) wheat Yangmai 16 using the primers specific to *TaCOMT-3D-Tnos* cassette. M, DL2, 000 DNA marker; P, the transformation vector pWMB-TaCOMT-3D as a positive control. **(C)** qRT-PCR analyses of the relative transcript levels of *TaCOMT-3D* in *TaCOMT-3D* transgenic lines at 7 d post inoculation (dpi) with *R*. *cerealis*. Three biological replicates per line were averaged and statistically treated (*t*-test; **P < 0.01). Bars indicate standard deviation of the mean. **(D)** Western blot pattern of the four *TaCOMT-3D*-overexpressing transgenic lines and WT Yangmai 16 using an anti-6 × His antibody. Similar results were obtained from three independent replicates. **(E)** Typical symptoms of sharp eyespot in the four *TaCOMT-3D*-overexpressing transgenic and WT Yangmai 16 plants.

The *TaCOMT-3D* overexpressing transgenic wheat lines were generated and used to further investigate the role of *TaCOMT-3D* in resistance response to *R*. *cerealis*. By using the primers specific to the transformation vector pWMB-TaCOMT-3D (Fig. 6A), PCR analysis results showed that the introduced *TaCOMT-3D* transgene was present in four wheat lines (OM1, OM2, OM3, and OM4) in T_0_-T_2_ generations (Fig. 6B), suggesting that the transgene could be inheritable in these four lines. qRT-PCR analyses indicated that the transcriptional levels of *TaCOMT-3D* in these four transgenic lines (OM1, OM2, OM3, and OM4) were markedly elevated than in wild- type (WT) Yangmai 16 (recipient) (Fig. 6C), and the introduced *TaCOMT-3D* was overexpressed in these four lines. The western blotting results proved that the introduced *TaCOMT-3D-His* was translated into the fusion protein in these four overexpressing lines, but not in WT Yangmai 16 (Fig. 6D). The sharp eyespot severity assessments in two successive (T_1_-T_2_) generations showed that compared with WT Yangmai 16, all these four *TaCOMT-3D-*overexpressing wheat lines (OM1, OM2, OM3, and OM4) displayed significantly enhanced resistance to sharp eyespot caused by *R*. *cerealis* R0301 (Fig. 6E, Table 1). For example, the average infection types and disease indices of these four *TaCOMT-3D*-overexpressing lines in T_2_ generation were 1.33, 1.34, 1.34, and 1.82, and 27.66, 26.70, 26.70, and 34.62, respectively, whereas the average infection type and disease index of WT Yangmai 16 were 2.08 and 41.54, respectively (Table 1). These results indicated that *TaCOMT-3D* overexpression improved resistance of the transgenic wheat to *R*. *cerealis* infection, and *TaCOMT-3D* positively participates in wheat resistance to *R*. *cerealis* infection.

**Table 1:** *Rhizoctonia cerealis* responses of *TaCOMT-3D* overexpressing transgenic and wild-type wheat lines.

Lines	T_1_		T_2_	
	Infection type	Disease index	Infection type	Disease index
OM1	1.34**	26.70**	1.82*	36.42*
OM2	1.33**	26.66**	1.44**	28.86**
OM3	1.67**	33.33**	1.40**	27.92**
OM4	1.34**	26.70**	1.34**	26.70**
WT	2.92	58.39	2.08	41.54

^*^or **significant difference between each transgenic lines and WT wheat at P < 0.05 or 0.01 (t-test).

OM1, OM2, OM3, and OM4 indicate *TaCOMT-3D*-overexpressing wheat lines; WT indicates untransformed wild-type Yangmai 16. *R*. *cerealis* isolate R0301 was used to infect plants in T_1_ and T_2_ generations. At least 30 plants in T_1_ and 110 plants in T_2_ generations for each line were assessed for disease intensity.

### *TaCOMT-3D* overexpression promotes lignin accumulation in stems of wheat

**Figure 7:**
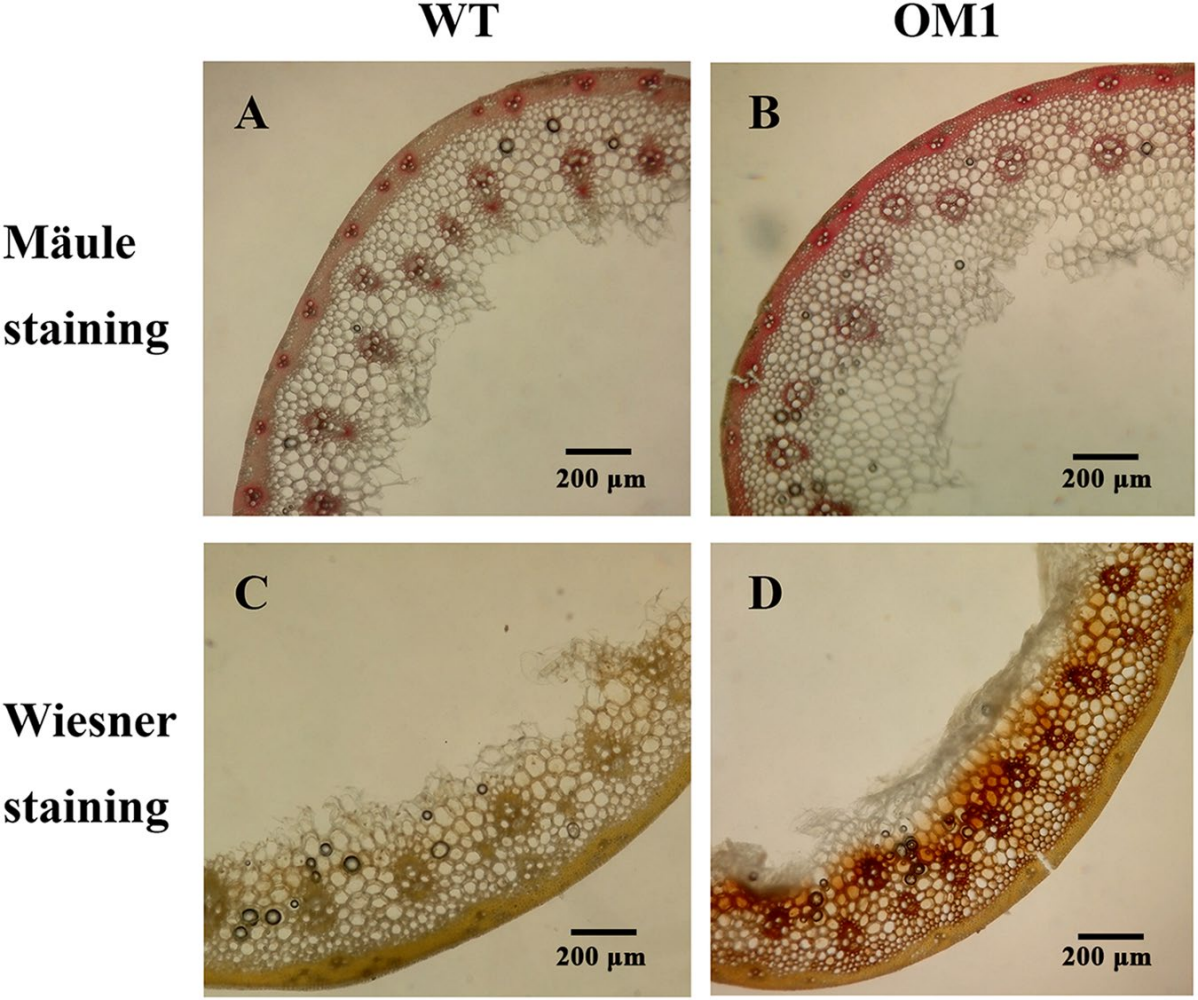
Lignin staining of *TaCOMT-3D*-overexpressing wheat plants and wild-type (WT) wheat Yangmai 16. (**A**,**B)** Transverse sections stained using the Wiesner method. **(C**,**D)** Transverse sections stained using the Mäule method. OM1, *TaCOMT-3D*-overexpressing transgenic line. Bar = 200 μm.

To explore if *TaCOMT-3D* affects the lignin biosynthesis in wheat, lignin content and composition in the second basal internode at milky stage were investigated. The examination data showed that the lignin content in *TaCOMT-3D-*overexpressing transgenic wheat lines was higher than that in WT Yangmai 16 plants (Table 2). Furthermore, hand-cut sections from the transgenic and WT plants were subjected to Wiesner and Mäule stains. When subjected to lignin-specific Wiesner reagent, cross sections of the *TaCOMT-3D-*overexpressing wheat lines displayed increased staining (red-brown) relative to the WT samples (Fig. 7A,B), which is indicative of a higher lignin level. When subjected to Mäule staining specific for syringyl monolignol, compared with WT samples, the *TaCOMT-3D*-overexpressing wheat lines displayed higher frequency of syringyl monolignol (Fig. 7C,D). These results suggested that *TaCOMT-3D* overexpression enhanced lignin biosynthesis, especially syringyl monolignol accumulation.

**Table 2:** Comparison of lignin content of basal second internodes in *TaCOMT-3D* overexpressing transgenic and wild-type wheat lines.

Lines	Lignin content (mg/g)	P value	Significance test
OM1	199.53 ± 11.01	0.048089108	*
OM2	227.06 ± 22.03	0.039334573	*
OM3	228.10 ± 21.30	0.035968636	*
OM4	218.75 ± 8.81	0.013258457	*
WT	172.79 ± 6.24	—	—

^*^Significant difference between each transgenic lines and WT wheat at P < 0.05 (t-test).

OM1, OM2, OM3, and OM4 indicate *TaCOMT-3D*-overexpressing wheat lines; WT indicates untransformed wild-type Yangmai 16.

### *TaCOMT-3D* overexpression enhances wheat stem mechanical strength

Previous study showed that *TaCM* expression participated in lignin synthesis and consequently contributed to stem strength and the lodging-resistant trait in wheat lodging-resistant line C6001 and lodging-susceptible wheat H4564^2^. Here, to evaluate the effect of TaCOMT-3D on stem lodging resistance in wheat, the stem mechanical (breaking) strength of the second basal internode of *TaCOMT-3D-*overexpressing and WT wheat Yangmai 16 lines was measured using a Texture Analyser (TA) with a three-point bend test setup at milky and harvest stages, respectively. As shown in Table 3, comparing with WT Yangmai 16 plants, these four *TaCOMT-3D-*overexpressing wheat lines (OM1, OM2, OM3, and OM4) exhibited significantly increased mechanical strength of the second basal internode at both milky and harvest stages. These data suggested that overexpression of *TaCOMT-3D* enhanced the stem mechanical strength of the transgenic wheat.

**Table 3:** Comparison of stem breaking strength of basal second internodes in *TaCOMT-3D* overexpressing transgenic wheat lines and wild-type wheat lines.

Lines	Milky stage		Harvest stage	
	Stem strength (Newton, N)	P value	Stem strength (Newton, N)	P value
OM1	11.85 ± 3.03*	0.045244	15.04 ± 3.51*	0.010972
OM2	15.38 ± 1.94*	0.043641	13.27 ± 3.87*	0.043407
OM3	14.08 ± 4.05*	0.032308	14.04 ± 3.05*	0.036096
OM4	14.89 ± 2.78**	0.002739	15.39 ± 3.61**	0.003181
WT	10.43 ± 1.78		10.44 ± 2.59	

^*^or **significant difference between each transgenic lines and WT wheat at P < 0.05 or 0.01 (t-test).

OM1, OM2, OM3, and OM4 indicate *TaCOMT-3D*-overexpressing wheat lines; WT indicates untransformed wild-type Yangmai 16.

## Discussion

Plant COMT catalyzes the multi-step methylation reactions of hydroxylated monomeric lignin precursors, consequently is involved in the lignin biosynthesis^13^. However, little is known about COMT participating in defense responses to necrotrophic fungal phytopathogens. In this study, the wheat caffeic acid O-methyltransferase encoding gene *TaCOMT-3D* was identified through microarray-based comparative transcriptomic analysis. The chromosome-based draft sequence alignment and PCR analysis indicated that *TaCOMT-3D* located on the long arm of wheat chromosome 3D. Previously, two genetic studies reported that two QTLs on 3D locating in the intervals *Xgwm61*~*Xgwm172–2*^40^ and *Xgwm314*~*Xgwm383*^41^ for sharp eyespot resistance, respectively, the latter of which explained 7% of the phenotypic variation^41^. Here, qRT-PCR analysis showed that the transcriptional level of *TaCOMT-3D* was higher in sharp eyespot-resistance wheat lines than in susceptible wheat lines/cultivars. To explain the differences in the expression profile of *TaCOMT-3D* between resistant and susceptible wheat, the gene promoter sequences were also cloned from CI12633 and Wenmai 6, respectively. However, no difference existed between the gene promoter sequences from these two wheat lines. Thus, we deduced the different expression profile of *TaCOMT-3D* between resistant and susceptible wheat may be due to the differences in regulatory pathways that activate/suppress *TaCOMT-3D* expression in wheat. The gene expression level was obviously elevated after infection of *R*. *ceraelis*. These results imply that *TaCOMT-3D* may participate in defense response of wheat to *R*. *cerealis* infection. BSMV-based VIGS technique has been shown to be an effective reverse genetic tool for rapidly investigating the defense functions of interesting genes in barley and wheat^42–46^. In this report, the BSMV-VIGS results show that silencing of *TaCOMT-3D* in the resistant wheat line CI12633 significantly impairs host resistance to *R*. *cerealis*. The data suggest that *TaCOMT-3D* is required for resistance response of wheat, at least the resistant wheat line CI12633, to *R*. *cerealis* infection. To further confirm the defense function of *TaCOMT-3D*, transgenic wheat plants overexpressing *TaCOMT-3D* were generated and characterized. Taken molecular assay together disease resistance assessments for T_0_-T_2_ generations, these results indicate that *TaCOMT-3D-*overexpressing transgenic wheat lines display significantly enhanced resistance to sharp eyespot during whole growth stages. Interestingly, *TaCOMT-3D* overexpression in wheat did not obviously affect major agronomic traits (including plant height, spike number, spike length, grain number per spike, and days to heading) in wheat (Supplemental Table S1). These results reveal that *TaCOMT-3D* positively contributes to host resistance to sharp eyespot caused by *R*. *cerealis* infection. To the best of our knowledge, this is the first report about a *COMT* that participates positively in plant resistance response to a necrotrophic fungal pathogen *R*. *cerealis*. However, the *TaCOMT-3D*-silencing and overexpressing wheat plants at seedling and adult stages showed typically susceptible phenotype to mixed strains (E9, E21, and E23) of *Blumeria graminis* f. sp. *tritici*, suggesting that *TaCOMT-3D* had no obvious effect on the wheat resistance phenotype to the mixed strains of powdery mildew. It is also important and interesting to further assay responses of *TaCOMT-3D* silenced and overexpressing wheat plants to infection of additional pathogens in future. Anyway, the current results broaden the current knowledge of plant defenses against pathogens.

The sequence alignment of amino acid sequences showed that the TaCOMT-3D protein shares a 98.03% identity with the wheat caffeic acid 3-O-methyltransferase TaCM^13^. Sequence analyses reveal that SAM binding motif, catalytic residues, and active site substrate binding/positioning residues of TaCOMT-3D are the same as those in TaCM, even though seven amino acid differences exist between the protein sequences of TaCOMT-3D and TaCM. Interestingly, TaCOMT-3D and TaCM proteins shared 100% identity in all the biochemically functional sites, including the SAM binding motif, catalytic residues and active site substrate binding/positioning residues. The previous study showed that TaCM protein exhibited a strong catalyzing activity towards caffeoyl aldehyde, caffeoyl-CoA, 5-hydroxyferuloyl-CoA and 5-hydroxyconiferaldehyde, revealing that TaCM is a typical COMT involved in lignin (syringyl monolignol) biosynthesis^13^. Additionally, among the functional sites, 13 amino acid residues being involved in the active site substrate binding/positioning residues have only one amino acid difference between TaCOMT-3D (M123, N124, L129, A155, H159, F165, F169, M173, H176, V309, I312, M313, and N317) and the alfalfa COMT (M123, N124, L129, A155, H159, F165, F169, M173, H176, V309, I312, M313, and N317; GenBank accession no. AAB46623)^32,47^, with V instead of I in the position 312. The crystal structure of COMT in complex with S-adenosyl-L-homocysteine, ferulic acid, and 5-hydroxyconiferaldehyde provide a structural understanding of the observed substrate preferences. And these crystal structures identify residues lining the active site surface that contact the substrates^32^. These results suggest that TaCOMT-3D should possess the COMT activity and may participate in lignin (especially syringyl monolignol) biosynthesis.

In previous studies, assays of knock-out/down mutants/transgenic plants indicated that COMT participated predominantly in syringl monolignol biosynthesis and can change lignin content and composition^12,36–39^. For example, the *Brachypodium Bd5139* mutant with a single nucleotide mutation in the caffeic acid *O*-methyltransferase encoding gene *BdCOMT6* displayed a moderately altered lignifiation in mature stems^38^. Ma and Xu reported that the expression of antisense *TaCM* in transgenic tobacco specifically reduced the COMT enzyme activity and marginally decreased lignin content, especially sharply reduced the syringl lignin monomer^13^. Ma reported that TaCM expression participated in lignin synthesis and was positively associated with stem strength and the lodging-resistant trait in wheat lodging-resistant line C6001 and lodging-susceptible wheat H4564^2^. However, little has been known about effect of COMT overexpression on lignin biosynthesis and stem mechanical strength in crop plants. Here, our results indicated that overexpression of *TaCOMT-3D* increased lignin level, especially syringyl monolignol content. Furthermore, *TaCOMT-3D* overexpression enhanced the basal stem mechanical strength, and the content of lignin and syringyl monolignol had a significantly positive correlation with the culm mechanical strength, which were in line with the effect of TaCM expression on lignin synthesis and stem strength in two wheat cultivars^2^. Previous studies documented that lignin accumulation and its composition (i.e., hydroxy-phenyl-, guaiacyl-, and syringyl-type monomers) are important factors influencing the breaking strength of wheat culm^2,3^. Zheng *et al*. showed that syringyl monolignol accumulation provided significant mechanical advantages to angiosperm species^3^. In this report, these assays suggest that TaCOMT-3D primarily enhances syringyl monolignol accumulation, leading to the increased the basal stem breaking strength. Lignin has been implicated in plant disease resistance^11,16,19^. Recently, the lignin biosynthesis-related enzyme ZmCCoAOMT2 in maize has been reported to confer quantitative resistance to southern leaf blight and gray leaf spot through increasing levels of lignin and other metabolites of the phenylpropanoid pathway^18^. Taken together, we can deduce that TaCOMT-3D enhances wheat defense response to *R*. *cerealis* and stem mechanical strength possibly through promoting lignin, especially syringyl monolignol accumulation.

In conclusion, the wheat gene *TaCOMT-3D* was identified through microarray-based comparative transcriptomics. The transcriptional level of *TaCOMT-3D* is higher in resistant wheat lines and significantly elevated after *R*. *cerealis* infection. *TaCOMT-3D* positively contributes to both resistance response to *R*. *cerealis* and adult stem mechanical strength possibly through promoting accumulation of lignin (especially syringyl monolignol). This study provides novel insights into the biological roles of COMT members in plants. *TaCOMT-3D* is a potential gene for improving resistance to both sharp eyespot and lodging.

## Materials and Methods

### Plant and fungal materials

Six wheat lines/cultivars, including sharp eyespot-resistant lines CI12633 and Shanhongmai, moderately-resistant cultivar Shannong 0431, moderately-susceptible cultivars Yangmai 158 and Yangmai16, and susceptible cultivar Wenmai 6, were used in this research.

A major Jiangsu virulent strain of the pathogen *R*. *cerealis* isolate R0301, was kindly provided by Profs. Huaigu Chen and Shibin Cai (Jiangsu Academy of Agricultural Sciences, China). A North China high-virulence strain of the pathogen *R*. *cerealis* isolate WK207 was provided by Prof. Jinfen Yu (Shandong Agricultural University, China).

### DNA or RNA extraction and cDNA synthesis

Genomic DNA for each sample was isolated from the wheat leaves using the CTAB method^48^.

Total RNA was extracted using TRIzol (Invitrogen, America), and then subjected to Rnase-free Dnase I (Promega, America) digestion and purification.

The purified RNA sample (2 µg) was reverse-transcribed to cDNA using the FastQuant RT Kit with gDNase (TransGen Biotech, China).

### Isolation and characterization of the *TaCOMT-3D* sequence

The microarray analysis using the Agilent wheat microarray indicated that a probe with Agilent GeneChip number A_99_P198406, corresponding to 3′-terminal sequence of a wheat cDNA sequence with accession number AK332908, was expressed at a significantly higher level in sharp eyespot-resistant wheat lines CI12633 and Shanghongmai than in the susceptible wheat Wenmai 6. This gene cloned from stems of the resistant wheat CI12633, based on AK332908 sequence, was designated as *TaCOMT-3D*. The 1319-bp full-length cDNA sequence of *TaCOMT-3D*was amplified by nested PCR from cDNA of CI12633 stems inoculated with *R*. *cerealis* R0301 for 4 d. The primers for the first round PCR were TaCOMT-3D-F1 (5′-GAAAGTAGGTCATCGCTCAGTC-3′) and AUAP (5′-GGCCACGCGTCG ACTAGTAC-3′). These for the second round PCR were TaCOMT-3D-F2 (5′-CTCGCTCACACTCAACGC-3′) and TaCOMT-3DL (5′-GGTGGATTTTA ATACACACAA-3′). The deduced protein sequence was analyzed by the Compute pI/Mw tool (http://web.expasy.org/compute_pi/) to determine the theoretical iso-electric point and molecular weight, Pfam database (http://pfam.xfam.org) and smart software (http://smart.embl-heidelberg.de/) to predict conserved motifs, DNAMAN software to analyze sequence identity.

### BSMV-VIGS assay for *TaCOMT-3D* function

To generate the BSMV:TaCOMT-3D recombinant construct, a 233-bp sequence specific to *TaCOMT-3D* (from 1022 to 1254 nucleotides in *TaCOMT-3D* cDNA sequence) was sub-cloned in an antisense orientation into the *Nhe* I restriction site of the RNAγ of BSMV (Fig. 5A). Following the protocols described by Holzberg *et al*.^49^, the tripartite cDNA chains of BSMV:TaCOMT-3D or the control BSMV:GFP virus genome were separately transcribed into RNAs, mixed, and used to inoculate the leaves of wheat CI12633 plants at the two-leaf stage. Then, the plants were grown in a 14 h light (22 °C)/10 h dark (12 °C) regime. To investigate if BSMV successfully infected CI12633 plants, and to test if *TaCOMT-3D* transcript was down-regulated, at 10 dpi with the BSMV virus, the fourth leaves of the inoculated seedlings were collected and subjected to analysis on the transcription of the BSMV coat protein gene (*CP*) by RT-PCR and the transcription of *TaCOMT-3D* by qRT-PCR. At elongation stage, the BSMV-infected CI12633 plants were further inoculated with *R*. *cerealis* isolate WK207 mycelia according to Wei *et al*.^50^. The infection types (ITs) of these plants were scored at 45 dpi with *R*. *cerealis* WK207 following the protocol described by Chen *et al*.^7^.

### *TaCOMT-3D*-overexpressing construct and transformation into wheat

The *TaCOMT-3D* ORF plus a 6 × His epitope tag sequence was sub-cloned into monocot transformation vector pWMB122^51^. In the resulting overexpression transformation vector pWMB-TaCOMT-3D (Fig. 6A), the transcript of the*TaCOMT-3D-His* fusion gene is driven by a maize ubiquitin (*Ubi*) promoter, and terminated by 3′-non-transcribed region of *Agrobacterium tumefaciens* nopaline synthase gene (*Tnos*). A total of 274 immature embryos of the wheat cultivar Yangmai 16 were transformed with through *Agrobacterium* harbouring the pWMB-TaCOMT-3D vector according to the protocol described by Wang *et al*.^52^.

### PCR detection of introduced *TaCOMT-3D* transgene

The presence of the *TaCOMT-3D-*overexpressing transgene in the transformed wheat plants was monitored by PCR using the specific primers, TaCOMT-3D-F (5′-GAGAGGTACGAGAGGGAGTT-3′, located in *TaCOMT-3D* coding sequence) and Nos-R (5′-TAAATGTATAATTGCGGGAC-3′, located in *Tnos* of the transformation vector). PCR was performed in a 25 μl volume containing ~200 ng genomic DNA, 12.5 μl 2 × Taq MasterMix (TransGen Biotech, China), 1 μl each primer (10 mΜ). The amplified product (318-bp size) specific to the introduced *TaCOMT-3D*-Tnos chimera was resolved on a 1.5% agarose gel and visualized by ethidium bromide staining.

### Analysis of transcriptional levels of target genes

qRT-PCR analysis with specific primers TaCOMT-3D-QF (5′-AGAAGGTGCCCTCGGGT-3′) and TaCOMT-3D-QR (5′-TGCATTGGCGTA GATGTAAGTG-3′) was used to investigate the relative transcriptional levels of *TaCOMT-3D* in various wheat plants. qRT-PCR was performed using SYBR Green I Master Mix (TaKaRa, Japan) in a volume of 25 μl on an ABI 7500 RT-PCR system (Applied Biosystems). Reactions were set up using the following thermal cycling profile: 95 °C for 15 min, followed by 40 cycles of 95 °C for 10 s, 56 °C for 30 s, and 72 °C for 32 s. The relative transcriptional levels of the target genes was calculated using the 2^−ΔΔCT^ method^53^, where the wheat *Actin* gene *TaActin* was used as the internal reference. The relative transcriptional levels of the tested genes in the *TaCOMT-3D*-overexpressing wheat lines or in BSMV:TaCOMT-3D-infected wheat plants were relative to those in WT recipient or in BSMV:GFP-infected wheat plants.

The transcriptional level of BSMV *CP* gene in wheat plants inoculated with BSMV, an indicator of BSMV infection, was investigated by semi-quantitative RT-PCR method with the BSMV-*CP* gene-specific primers (BSMV-CP-F: 5′-TGACTGCTAAGGGTGGAGGA-3′, BSMV-CP-R: 5′-CGGTTGAACATCACGAAGAGT-3′).

### Western blotting analysis for TaCOMT-3D-overexpressing protein

The TaCOMT-3D-His fusion protein in the overexpressing wheat lines was visualized via western blotting analysis. Total proteins were extracted from ~0.2 g of stems inoculated with *R*. *cerealis* R0301 for 20 d by using the tissue protein extraction kit (CWBIO, China). Total soluble proteins (~20 μg) for each line were separated on 12% SDS-PAGE and transferred to polyvinyl difluoride membranes (Amersham). The blotting membranes were incubated with 2500-fold diluted Anti-His Mouse Monoclonal Antibody (TransGen Biotech, China) at 4 °C overnight, then incubated with 4, 000-fold diluted Goat Anti-Mouse IgG (H + L), HPR conjugated secondary antibody (TransGen Biotech, China) at 22–23 °C for 1 h. The TaCOMT-3D-His fusion protein was visualized using the Pro-light HRP Chemiluminescent Kit (TransGen Biotech, China).

### Assessment of response of transgenic wheat plants to *R*. *cerealis*

At the active tillering stage, at least 30 plants in T_1_ and 110 plants in T_2_ generations for each line of the *TaCOMT-3D*-overexpressing wheat lines and non-transgenic wheat Yangmai 16 (recipient) were inoculated with sterilized grains harboring the well-developed mycelia of *R*. *cerealis* isolate R0301 following the protocol of Zhu *et al*.^54^. According to the modified protocol described by Chen *et al*.^7^, at harvest stage (about 60 dpi), the infection type (IT) of each plant was scored based on the disease lesion squares^54^, and disease index (DI) for each wheat line were categorized.

### Examination of stem mechanical strength

Stem strength testing of the material was carried out using a Texture Analyser (TA) with a three-point bend test setup as described by Miller *et al*.^55^. Briefly, the TA was fitted with a load cell with maximum loading capacity of 5 kg. The support stands were set at 2.5 cm apart (across which the 5 cm stem sample was placed) and the testing probe was set to move at a constant speed of 2 mm/s. The TA, connected to a computer, produces a real-time graphical output, representing the mechanical profile of the stem sample being tested. From this graph, F _max_, the absolute resistance of the stem sample to break under-load, were obtained.

### Examination of lignin content and syringyl monolignol

Lignin content was quantitatively measured by using the lignin ELISA Kit (ChemFaces, China) according to the manufacturer’s protocols. For histochemical analysis, fresh hand-cut sections were prepared from basal second internodes of wheat. Wiesner and Mäule staining methods were performed as previously described^26^. Briefly, Wiesner staining was performed by incubating sections in 1% phloroglucinol in ethanol: water (7:3) with 30% HCl. Mäule staining was performed by first incubating sections in KMnO_4_. After 10 min, sections were washed and acidified with HCl for 1 min, washed again, and then incubated in NaHCO_3_. Cross sections were immediately observed under an optical microscope.

## Electronic supplementary material

Supplementary Information
