# Supplementary material for: A wheat caffeic acid 3-O-methyltransferase TaCOMT-3D positively contributes to both resistance to sharp eyespot disease and stem mechanical strength
Source: Sci Rep. 2018 Apr 25;8:6543. doi: 10.1038/s41598-018-24884-0 (PMC5916939; doi:10.1038/s41598-018-24884-0)
Supplement: Supplementary file 1 — Supplementary Information [file 41598_2018_24884_MOESM1_ESM.docx]

**Title: A wheat caffeic acid 3-O-methyltransferase TaCOMT-3D positively contributes to both resistance to sharp eyespot disease and stem mechanical strength**

**Authors:** Minxia Wang^1^**†**, Xiuliang Zhu^1^**†**, Ke Wang^1^, Chungui Lu^2^, Meiying Luo^1^, Tianlei Shan^1^, Zengyan Zhang^1^*

**Institutions:** 1 The National Key Facility for Crop Gene Resources and Genetic Improvement, Institute of Crop Science, Chinese Academy of Agricultural Sciences, Beijing 100081, P.R. China; 2 School of Animal, Rural and Environmental Sciences, Nottingham Trent University, Brackenhurst Campus, Nottingham NG250QF, United Kingdom

**†** Authors contributed equally to this work

***Corresponding author:** Zengyan Zhang; Telephone: +86-10-82108781;

Fax: +86-10-82105819; E-mail: [zhangzengyan@caas.cn](mailto:zhangzengyan@caas.cn)

**Supplementary data**

**Supplemental Figure S1 The monolignol biosynthesis pathway, adapted from previous studies (Boerjan et al., 2003; Bhuiyan et al., 2009; Trabucco et al., 2013).** 4CL, 4-hydroxycinnamoyl-CoA ligase; C3H, p-coumarate 3-hydroxylase; C4H, cinnamate 4-hydroxylase; CAD, cinnamylalcohol dehydrogenase; CCoAOMT, caffeoyl-CoA O-methyltransferase; CCR, cinnamoylCoA reductase; COMT, caffeic/5-hydroxyferulic acid O-methyltransferase; F5H, ferulate 5-hydroxylase; HCT, hydroxycinnamoyltransferase; PAL, phenylalanine ammonia lyase.


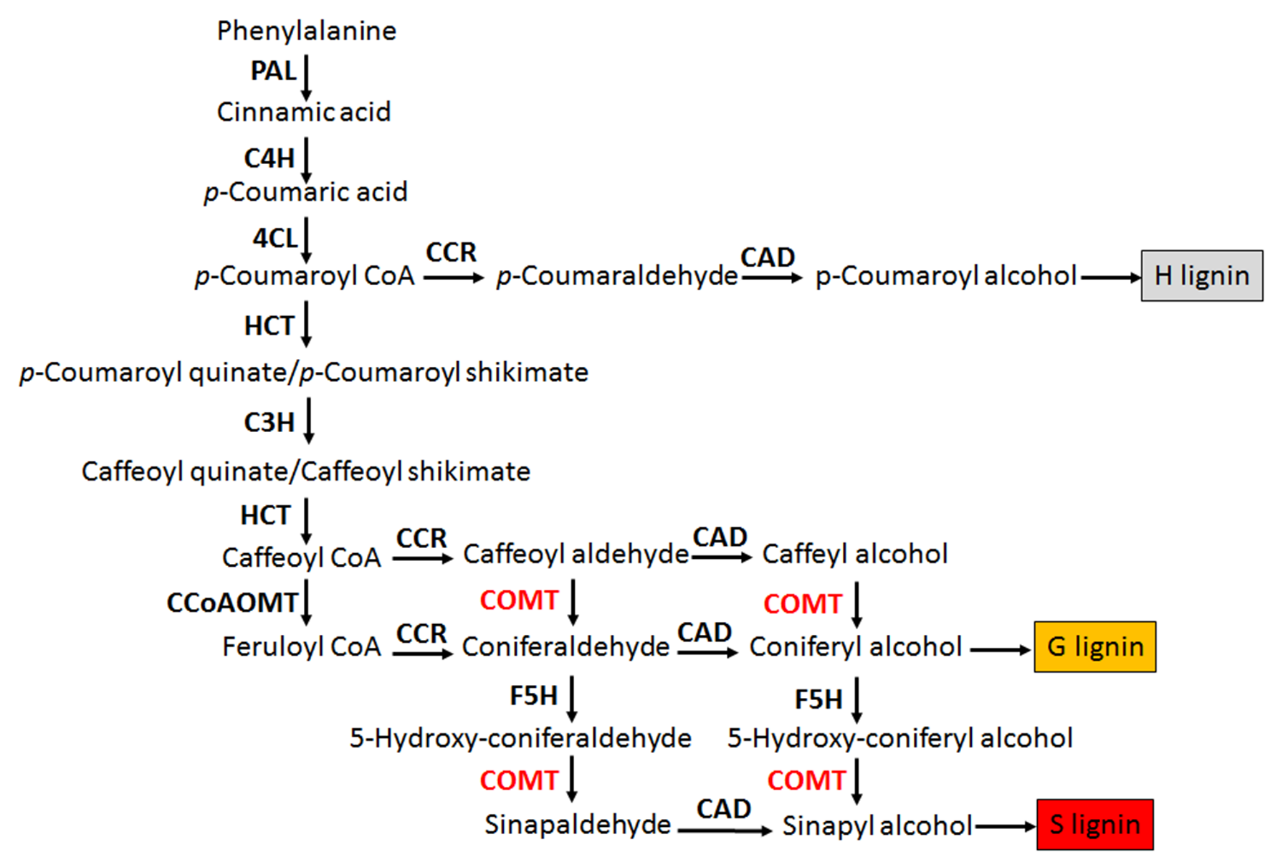


**Supplemental Figure S2 Alignment of promoter sequences of *TaCOMT-3D* in resistant wheat line CI12633 and susceptible wheat cultivar Wenmai 6.** The software DANMAN was used to perform the sequence alignment.


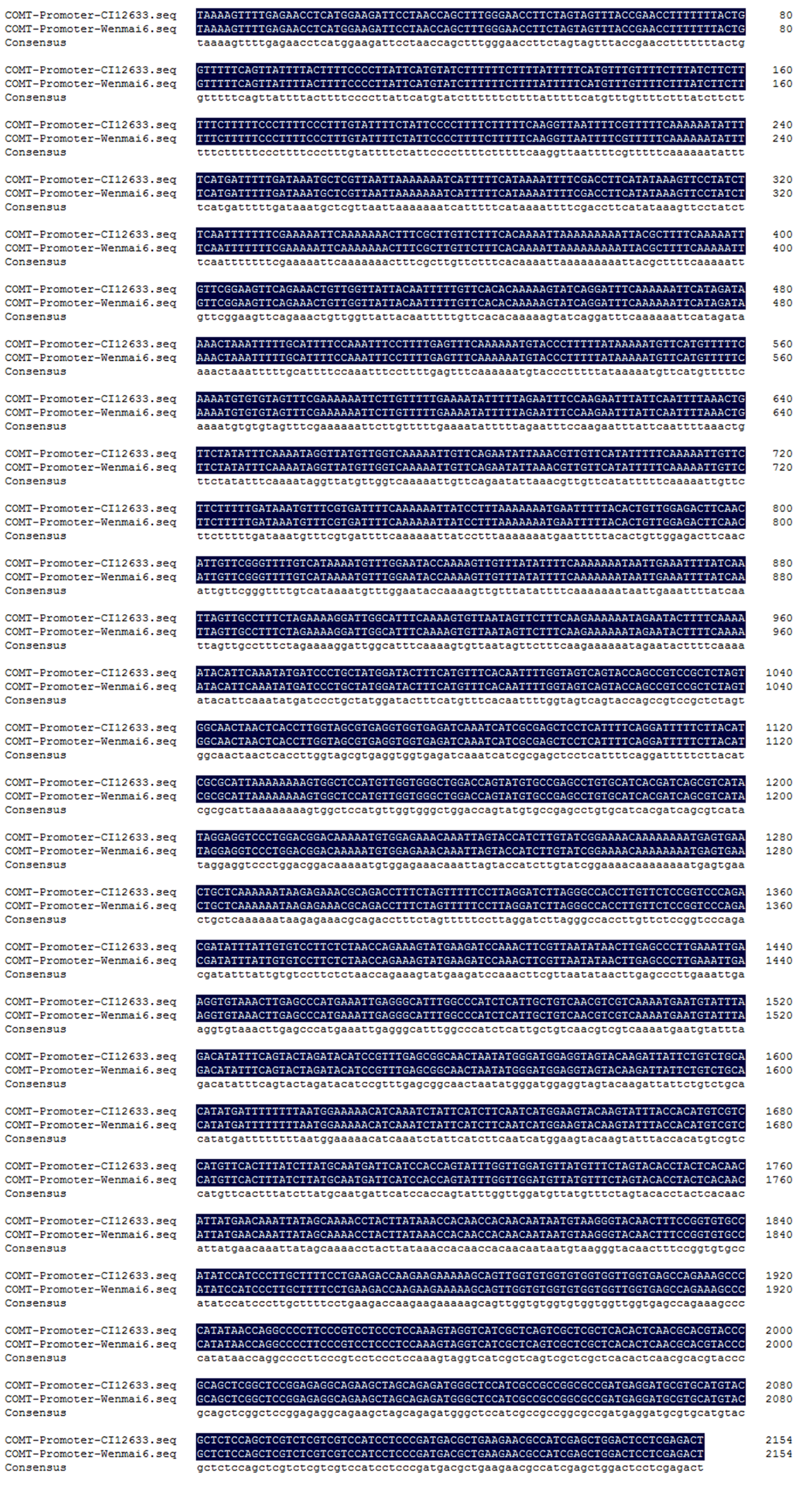


**Supplemental Table S1** Major agronomic traits in *TaCOMT-3D* overexpressing transgenic wheat lines and wild-type wheat lines

| Lines | Plant height (cm) | Spike number | Spike length (cm) | Grain  number | Days to heading (days) |
| --- | --- | --- | --- | --- | --- |
| OM1 | 69.43 | 2.87 | 8.67 | 35.33 | 83.17 |
| OM2 | 69.23 | 2.68 | 8.60 | 33.67 | 83.07 |
| OM3 | 72.55 | 2.67 | 8.98 | 34.67 | 83.00 |
| OM4 | 70.57 | 3.67 | 9.37 | 35.67 | 82.87 |
| WT | 71.33 | 2.70 | 8.77 | 34.67 | 83.17 |

When harvested in greenhouse, the major agronomic traits of wheat lines were measured. The values are the average of plants investigated. OM1, OM2, OM3, and OM4 indicate *TaCOMT-3D*-overexpressing wheat lines; WT indicates untransformed wild-type Yangmai 16.
